# Proteomic Analyses of Early *Versus* Advanced Primary Open-Angle Glaucoma Uncover Biochemical Signatures Associated With Disease Staging

**DOI:** 10.1016/j.mcpro.2026.101625

**Published:** 2026-07-18

**Authors:** Tianwei Ellen Zhou, David J. Mathew, Karen Wigg, Darren Chan, Jenny Wanyu Zhang, Carmen Balian, Irfan N. Kherani, Matthew B. Schlenker, Igor Jurisica, Jeremy M. Sivak

**Affiliations:** 1 Department of Ophthalmology and Vision Sciences, University of Toronto, Toronto, Ontario, Canada; 2 Department of Ophthalmology, Queen’s University, Kingston, Ontario, Canada; 3 Donald K. Johnson Eye Institute, University Health Network, Toronto, Ontario, Canada; 4 Kensington Health Research Institute, Toronto, Ontario, Canada; 5 Laboratory Medicine and Pathobiology, University of Toronto, Toronto, Ontario, Canada; 6 Osteoarthritis Research Program, Schroeder Arthritis Institute, University Health Network, Toronto, Ontario, Canada; 7 Data Science Discovery Centre for Chronic Diseases, Krembil Research Institute, University Health Network, Toronto, Ontario, Canada; 8 Institute of Neuroimmunology, Slovak Academy of Sciences, Bratislava, Slovakia; 9 Biological and Life Sciences Division, School of Digital Public Health, Mohamed bin Zayed University of Artificial Intelligence, Abu Dhabi, UAE

**Keywords:** glaucoma, proteomics, aqueous humor, inflammation, TRPC6, disease staging

## Abstract

Primary open-angle glaucoma (POAG) is a leading cause of blindness, yet the biochemical mechanisms underlying disease progression remain poorly understood. This study characterized aqueous humor (AH) across early and advanced stages of POAG using large-scale proteomics. AH samples were collected from patients with mild POAG, advanced POAG, and non-glaucomatous controls in this prospective cross-sectional study. Profiling of 8000 proteins was performed using an antibody array. Hierarchical clustering, principal component analysis (PCA), Gene Ontology (GO), and pathway enrichment analyses were performed to identify differentially expressed proteins, biological processes, and molecular functions associated with disease stages. Top differential proteins were also correlated with key clinical parameters. Proteomic signatures demonstrated distinct clustering between control and glaucoma patients, with progressive shifts from mild to advanced stages. Comparisons between control and each glaucoma group revealed significant enrichment in pathways related to wound healing, extracellular matrix (ECM) remodeling, and immune responses. Notably, distinct proinflammatory and immune proteins and pathways were enriched when comparing mild to advanced POAG stages. Finally, the top differential AH proteins correlated with multiple clinical endpoints, including intraocular pressure and a variety of retinal outcomes. This signature revealed a tight network associated with disease stage, oriented on immune cell migration and function, and identifying soluble TRPC6 as a novel biomarker. To our knowledge this is the first study to compare large scale proteomic analysis of AH from patients with POAG across disease stages, suggesting new mechanisms underlying glaucoma progression.

## Highlights

• Biochemical changes in progressive primary open-angle glaucoma (POAG) are unclear.
• Performed proteomic profiling of aqueous humor from control, early, or advanced POAG.
• Signatures clustered between control and POAG, and from mild to advanced stages.
• Highlighted pathways were enriched in markers of inflammation and immune function.
• Top hits associated with clinical retinal outcomes, identifying TRPC6 as a biomarker.

## Graphical Abstract

**Figure:**
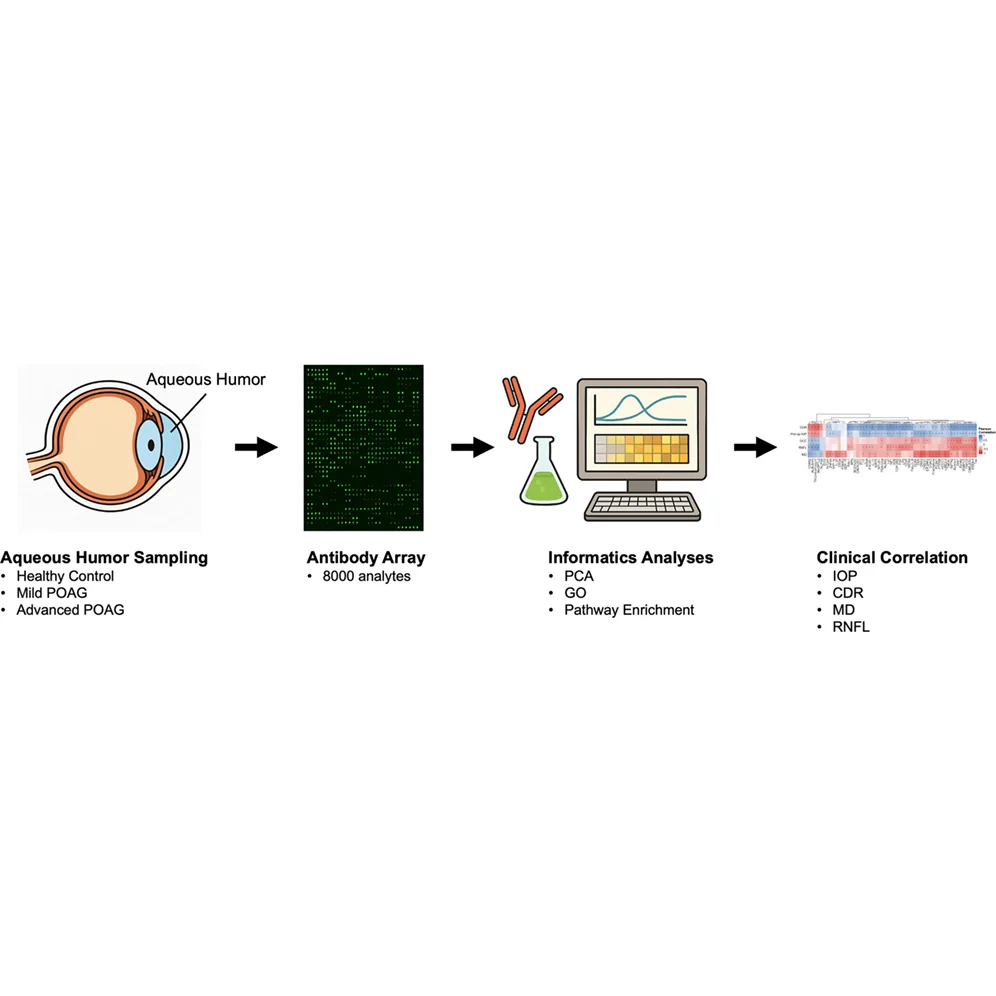

## In Brief

Large-scale proteomic profiling was performed on aqueous humor from early and advanced stages of primary open-angle glaucoma, or controls, using an antibody array. The resulting signatures demonstrated a shift towards distinct proinflammatory and immune signals with advanced disease. Top differential proteins were also associated with clinical disease measures of retinal degeneration, identifying TRPC6 levels as a highly regulated biomarker.

Glaucoma is a leading cause of blindness globally, characterized by progressive loss of retinal ganglion cells that cause irreversible visual deficiency (1). With an aging population, 111.8 million people are estimated to be affected by glaucoma by 2040 (2). Primary open-angle glaucoma (POAG) is the most common type with an estimated current global population of 68.56 million (3). In North America, POAG accounts for 85% to 90% of all cases (4, 5), representing a major health care burden.

The pathogenesis of POAG is complex, with both intraocular pressure (IOP)-related and IOP-independent mechanisms implicated. Ocular hypertension (elevated IOP) is the most prominent risk factor for POAG and its progression (6, 7), and is most commonly believed to be a result of reduced aqueous outflow, which in turn, is a product of trabecular meshwork (TM) blockage or dysfunction (8). Meanwhile, a proportion of patients that develop glaucoma continue to worsen despite low IOPs. In this case, a variety of IOP-independent mechanisms, including extracellular matrix dysfunction, failure of cellular repair mechanisms, and inflammation, are thought to contribute a network of additional etiopathogenic factors (9, 10, 11). Of note, the official staging method for early, moderate or advanced glaucoma is solely based on clinical parameters, namely; the cup-to-disc ratio (CDR) and visual field defects (12).

In the past 2 decades, there have been numerous studies that analyzed the proteomic or metabolomic signature of aqueous humor (AH) in glaucoma patients as a minimally invasive method of direct sampling near the site of TM dysfunction. However, these studies have generally focused on a single stage of glaucoma (13, 14, 15, 16, 17). In doing so, they only provided a “snapshot” of the biochemical profile at a certain stage (usually advanced), rather than identifying distinct proteomic profiles at different disease stages. In addition, most biochemical studies have used multiplex profiling to assess only a targeted list of proinflammatory cytokines or chemokines (18, 19, 20, 21), rather than offering a less biased analyses of a large portion of the total proteome. Together, these aspects of the current literature leave an important knowledge gap concerning the differential proteomic signatures associated with glaucoma staging from early to advanced disease.

Given this issue, the goal of the present pilot study was to explore the proteomic profiles of different stages of POAG in patient AH samples. To cast a wide net, we performed large-scale proteomic analyses of AH from patients with either mild or advanced POAG in comparison to non-glaucomatous control samples. This approach allowed us to better understand the corresponding biochemical signatures and uncover changes in key proteins and pathways associated with disease staging.

## Experimental Procedures

### Patients

This prospective comparative study was approved by the University Health Network and University of Toronto Research Ethics Boards (#22-5003 and 42228, respectively), and conformed to the tenets of the Declaration of Helsinki. Glaucoma patients scheduled for surgery at the Toronto Western Hospital or Kensington Eye Institute were introduced to the study, and those who provided informed consent to participate were enrolled. Patient data were collected and stored in accordance with applicable privacy regulations and were de-identified prior to analysis using a randomized sample ID. Inclusion criteria were: Participants were above 18 years of age, able to provide consent, and meeting surgical criteria for cataract, glaucoma, or combined surgeries (as determined by their ophthalmologists). For the non-glaucoma group; normal visual field, cup-to-disc ratio <0.5, normal and symmetrical retinal nerve fiber layer (RNFL) thickness on optical coherence tomography (OCT). For the glaucoma groups; meeting the diagnostic criteria for mild or advanced POAG as defined below (*Glaucoma Staging*). Subjects were excluded if they did not meet the inclusion criteria, or dad a diagnosis of uveitis, diabetic retinopathy, wet macular degeneration, or systemic inflammatory or autoimmune disease. Patient demographics and clinical data were recorded. These included age, sex, race, systemic conditions, oral medications, glaucoma medications and duration of use, IOP prior to surgery, CDR, visual field parameters, and retinal nerve fiber layer (RNFL) thickness on optical coherence tomography (OCT). Race/ethnicity were self-reported and collected from electronic health records at enrollment using standardized categories (White, Black or African American, Asian, Hispanic or Latino, and Other).

### Glaucoma Staging

Glaucoma staging was based on the mean deviation (MD) values of a visual field test performed within 6 months of study enrollment: for early glaucoma, a MD no worse than −6 dB (dB), and advanced glaucoma, MD worse than −12 dB (12, 22).

### Sample Size

A sample size calculation was performed. The effect size estimation was based on existing literature on cytokine profiling in POAG patients, estimated to be 8 to 12 patients per group, with an α of 0.05 and power set to 80% (15, 23, 24). A two-sided test was used.

### Sample Collection

From each eye, 100 μl of AH was carefully collected using a 30-gauge needle mounted on a 1-mL syringe, introduced into the anterior chamber at the limbus prior to any surgical intraocular entry. The samples were snap frozen on dry ice, aliquoted, and stored at −80 °C until analyses, as previously described (23, 24, 25).

### Sample Analyses

The AH samples were thawed and submitted to quantitative proteomic analysis using the RayBiotech Human L-8000 Glass Slide Array, according to the manufacturer’s direction (RayBio). Relative expressions of all samples and targets are attached as intensity-normalized scores in Supplemental Table S1. After co-normalization, batch correction and additional quality control, the top 500 most variable proteins were included in a hierarchical cluster analysis of AH proteins. Differentially expressed proteins among the three groups were identified using thresholds of log_2_ fold change >0.25 or < −0.25, and –log_10_(*p*-value) > 1.2 (approximately *p* < 0.063). Clustering was performed in R (26), and final cluster assignments were derived using the hclust function due to its strong concordance with consensus clusters and the advantage of a more accessible, repeatable, and interpretable algorithm.

### Gene Ontology and Pathway Enrichment Analyses

Gene Ontology (GO) enrichment analysis was performed using clusterProfiler (27) to identify significantly enriched biological processes (BP), molecular functions (MF), and cellular components (CC) among the input gene lists. To determine fold change between groups, for each protein, the distribution was assessed using the Shapiro-Wilk test. If both groups being compared yielded Shapiro-Wilk *p*-values <0.1, the non-parametric Wilcoxon rank sum test was used instead of the *t* test. To further consolidate and interpret proteomic changes at the pathway level, integrated pathway enrichment analysis was performed using pathDIP version 5.0.21.10 (28) (https://ophid.utoronto.ca/pathDIP). This platform incorporates both curated and extended protein–pathway associations across 12 major pathway databases (ACSN2, BioCarta, HumanCyc, KEGG, Panther, PathBank, Reactome, PharmaGKB, RECON3D, SIGNOR 3.0, UniProt, and WikiPathways). Pathway enrichment was evaluated using a Benjamini–Hochberg false discovery rate (FDR) correction, with pathways considered significant at q < 0.05. Predicted pathway associations were filtered at a minimum confidence score of 0.99 based on integrated protein–protein interaction (PPI) data from the IID (Integrated Interactions Database; https://ophid.utoronto.ca/iid (29)). The resulting data provided consolidated pathways and biological processes underlying glaucoma staging.

### Human Tissue Staining

Healthy and glaucomatous human donor eyes were obtained and prepared for immunostaining from the local Human Eye Biobank, according to a protocol approved by our institutional ethical review board, and in accordance with the declaration of Helsinki, as previously reported (25, 30). Briefly, eye tissues were fixed in 4% PFA before embedding in OCT for cryosectioning. For staining, sections were briefly rinsed with PBS and permeabilized with 0.1% Triton X-100 in PBS (tPBS) for 10 min. Non-specific binding was blocked with 5% serum in tPBS 1 h. Primary antibodies raised against TRPC6 (Alomone Labs, #ACC-017), and RBPMS (PhosphoSolutions, #1832-RBPMS), were diluted with 1% serum and applied to the sections overnight at 4C. Sections were then rinsed with tPBS for three x 10 min. Secondary antibodies were made up with tPBS and incubated for 1 h at room temperature, and then rinsed with tPBS for three x 10 min. Sections were stained with DAPI for 10 min, and then rinsed and mounted for imaging on a Nikon Eclipse Ti confocal microscope.

## Results

### Matched Patient Demographics and Clinical Data

Ten (10) patients were originally recruited for each group, with an effort made to match the associated demographics. Note that three patients were originally enrolled in the mild glaucoma group. However, their glaucoma progressed rapidly to the advanced stage by the time of the surgery and upon a thorough chart review they were reclassified for our analyses. After this chart review one patient originally enrolled in the advanced glaucoma group was reclassified as mild. The average ages for the control group (control), the mild glaucoma group (MG) and the advanced glaucoma group (AG) were 69.0 ± 10.4, 72.4 ± 7.6 and 72.5 ± 8.8 year-old (yo), respectively (Table 1). The control group had six females and four males; the MG group had five females and three males whereas the AG group had seven females and five males. The control and MG groups had equal numbers of left and right eyes. The AG group had seven right eyes and five left eyes.

**Table 1:** Demographics and glaucoma characteristics

Characteristic	Control	Mild glaucoma (MG)	Advanced glaucoma (AG)	*p* Values
Age (year-old)	69.0 ± 10.4	72.4 ± 7.6	72.5 ± 8.8	All: NS
Sex (Female: Male)	6 : 4	5 : 3	7 : 5	All: NS
Laterality (Right: Left)	5 : 5	4 : 4	7 : 5	All: NS
Race/Ethnicity (n) (White/Asian/Black)	9/1/0	7/1/0	10/0/2	All: NS
BCVA	0.25 ± 0.27	0.29 ± 0.31	0.53 ± 0.44	All: NS
Pre-operative IOP (mmHg)	12.1 ± 2.1	15.0 ± 4.6	19.0 ± 5.5	Control *versus* Advanced: *p* = 0.0031
CDR	0.43 ± 0.14	0.78 ± 0.08	0.80 ± 0.13	Control *versus* Mild: *p* < 0.0001 Control *versus* Advanced: *p* < 0.0001 Mild *versus* Advanced: NS
MD (dB)	−1.01 ± 2.51	−3.38 ± 2.65	−13.61 ± 7.00	Control *versus* Mild: NS Control *versus* Advanced: *p* < 0.0001 Mild *versus* Advanced: *p* = 0.0003
RNFL thickness (μm)	86.29 ± 11.94	65.50 ± 10.81	62.83 ± 9.93	Control *versus* Mild: *p* = 0.0030 Control *versus* Advanced: *p* = 0.0004 Mild *versus* Advanced: NS
GCC thickness (μm)	72.71 ± 5.36	62.13 ± 5.25	55.00 ± 10.18	Control *versus* Mild: *p* = 0.0389 Control *versus* Advanced: *p* = 0.0004 Mild *versus* Advanced: NS

BCVA, best correlated visual acuity; CDR, cup-to-disc ratio; dB, decibel; GCC, ganglion cell; MD, mean deviation; RNFL, retinal nerve fiber layer complex.

**Figure S1:**
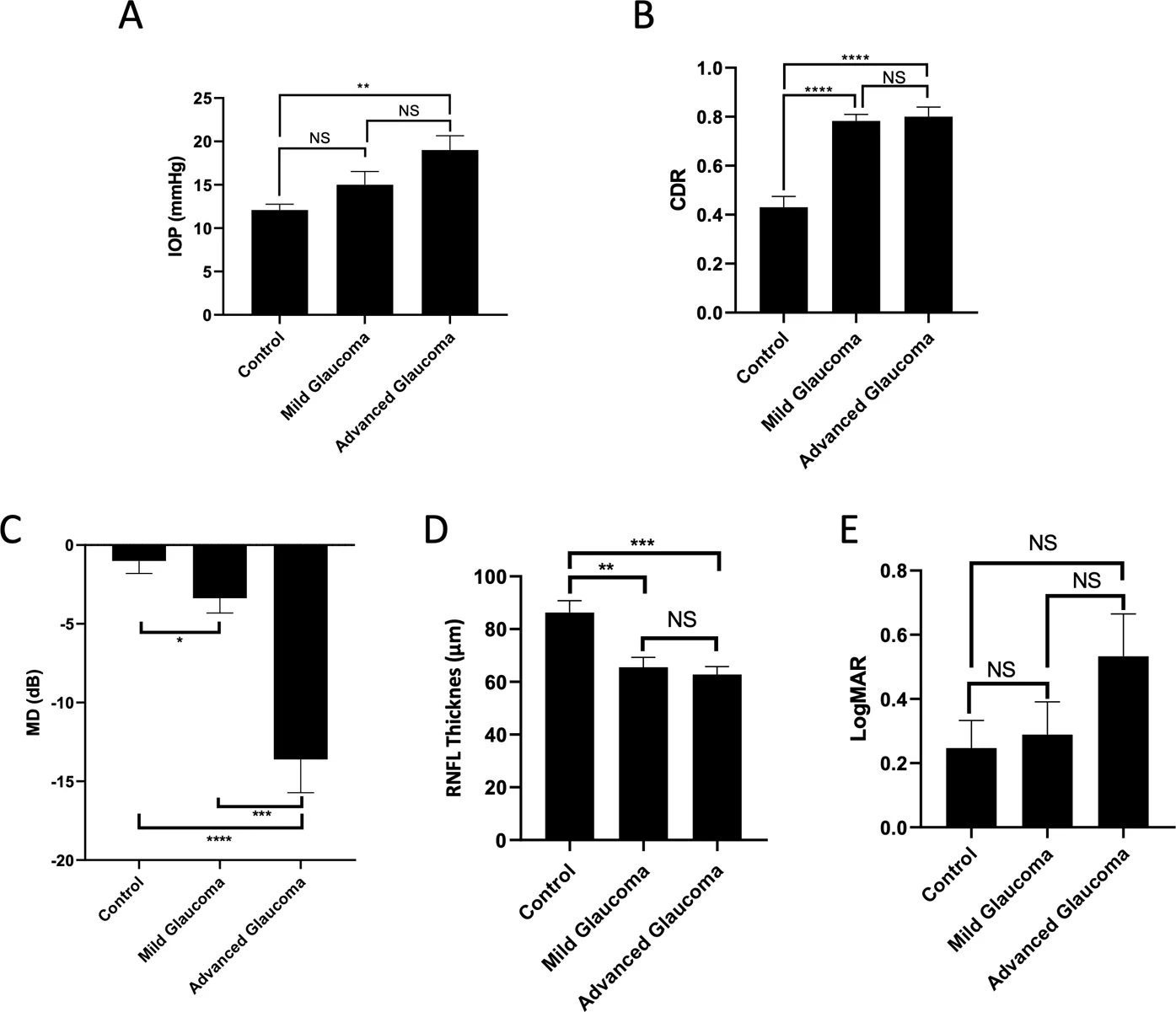
**Analyses of key clinical parameters.***A,* preoperative IOPs, B) CDR, C) MD, D) RNFL thickness, and E) LogMAR. (∗*p* < 0.05, ∗∗*p* < 0.01, ∗∗∗*p* < 0.0005, and ∗∗∗∗*p* < 0.001., bars are SE). CDR, cup-to-disc ratio; IOP, intraocular pressure; MD, mean deviation; RNFL, retinal nerve fiber layer.

Compared to the control group, MG and AG groups exhibited significantly increasing evidence of disease and visual field loss (Table 1, Supplemental Fig. S1). The best-corrected visual acuities (BCVA, logMAR) were: 0.25 ± 0.27 (control), 0.29 ± 0.31 (MG) and 0.53 ± 0.44 (AG), respectively. The corresponding pre-operative IOPs increased from 12.1 ± 2.1 mmHg (control), to 15.0 ± 4.6 (MG) and 19.0 ± 5.5 mHg (AG). Similarly, the cup-to-disk ratios (CDRs) increased from 0.43 ± 0.14 (control), to 0.78 ± 0.08 (MG) and 0.80 ± 0.13 (AG). The mean deviations (MDs) of the visual fields were −1.01 ± 2.51 dB (control), −3.38 ± 2.65 dB (MG) and −13.61 ± 7.00 dB (AG). The RNFL thicknesses reduced from 86.29 ± 11.94 μm (control), to 65.50 ± 10.81 μm (MG) and 62.83 ± 9.93 μm (AG). Similarly, the ganglion cell complex (GCC) thicknesses reduced from 72.71 ± 5.36 μm (control) to 62.13 ± 5.25 μm (MG) and 55.00 ± 10.18 μm (AG).

### Initial Proteomic Data Analysis

**Fig. 1:**
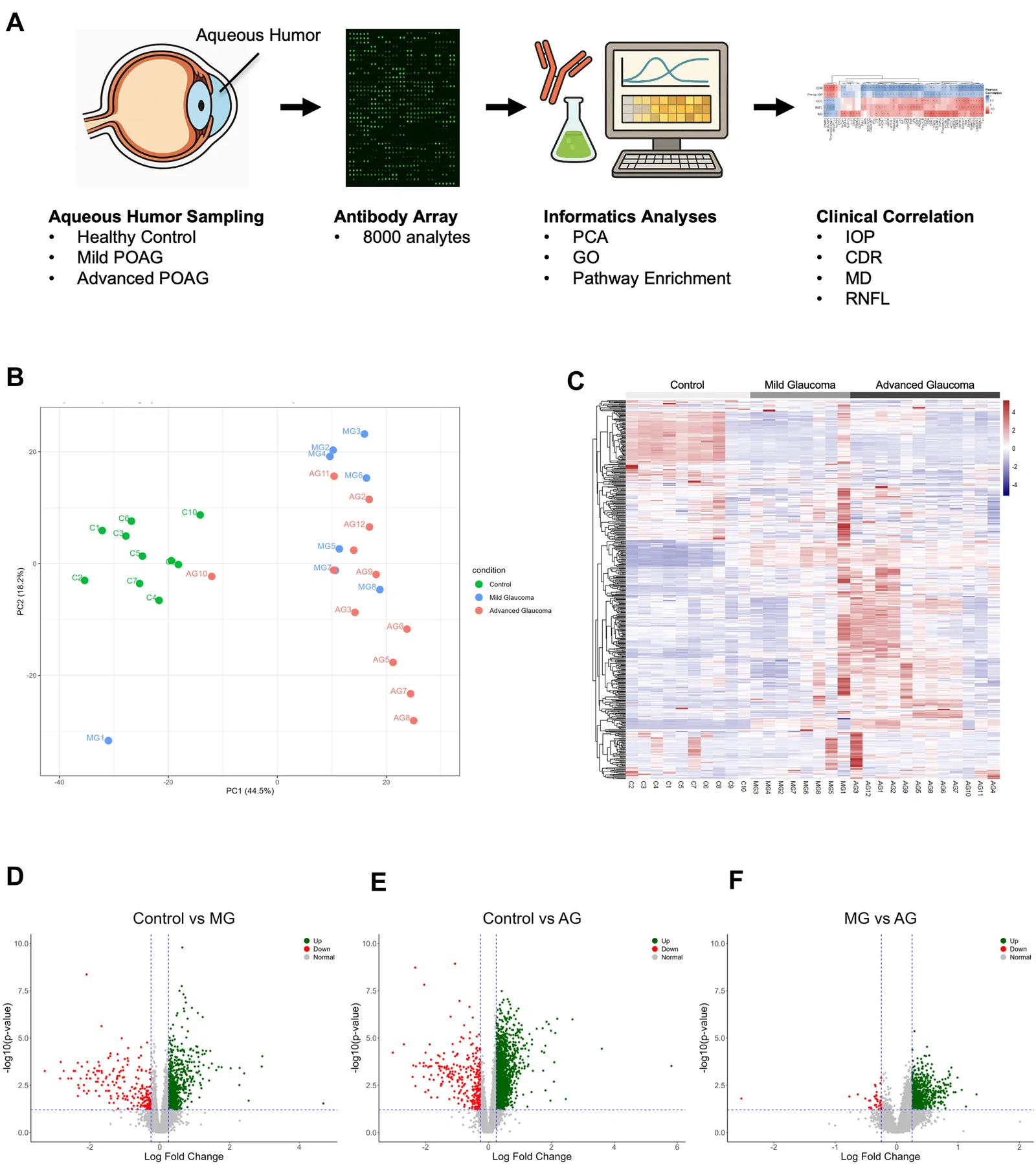
**Proteomic data clustering and analyses reveal broad distinctions between POAG stages and identify altered proteins.***A*, a schematic overview of the experimental design and analyses. *B*, PCA analyses indicate a broad distinction between control (*green*) and POAG samples along PC1. MG (*blue*) and AG (*red*) are clustered in a more nuanced way along PC2. *C*, Similarly, HCA of the top 500 most variable proteins across the groups indicates consistent differences between the three groups. *D*, Volcano plot highlighting significantly upregulated (*green*) and significantly downregulated (*red*) proteins in a comparison between control and MG groups. *E*, Volcano plot comparing proteins altered between control and AG groups. *F*, Volcano plot comparing proteins altered between MG and AG groups.

As detailed in the methods, AH samples collected from each patient were subjected to large-scale proteomic analyses of 8000 proteins using the Raybiotech L-8000 array (Fig. 1*A*). We first evaluated data quality across the three groups to ensure the robustness of our results. Principal Component Analysis (PCA) demonstrated a clear separation between the controls (green) and glaucoma samples (MG in blue and AG in red) along PC1, reflecting a substantial biochemical difference between control and glaucoma patients (Fig. 1*B*). The distinction between samples from MG and AG stages was more nuanced, but they tended to cluster separately along PC2 (Fig. 1*B*). Notably, two potential biochemical outliers, one from the AG group and one from the MG group, were identified. However, for completeness and transparency, these samples were included in the subsequent analyses.

Next, we identified the top 500 most variable proteins across the groups and used them to perform hierarchical clustering analysis (HCA). This method visually represents differential protein levels across the three groups (Fig. 1*C*). HCA demonstrated distinct clusters between the groups, underscoring broad shifts in proteomic signatures at different stages of POAG, moving from control to AG. To comprehensively investigate the altered concentration of specific markers in different stages of glaucoma, we identified differential proteins by applying thresholds of log_2_ fold change >0.25 or < −0.25 and –log_10_(*p*-value) > 1.2, resulting in a total of 990 altered proteins in the MG group compared to the control group, 2674 altered proteins in the AG group compared to the control group and 828 altered proteins in the AG groups compared to the MG group (Fig. 1, *D–F*, full lists in Supplemental Table S2).

### Identification of Top Altered Proteins Between Different Stages of Glaucoma

From our differential analyses, we highlighted the top significantly altered proteins for each comparison, ranked by fold change. These results are presented for the MG group compared to the control group (Table 2), the AG group compared to the control group (Table 3), and the AG group compared to the MG group (Table 4), highlighting specific proteins that were significantly altered between POAG stages. Note: the full lists of differential proteins for each comparison are available in Supplemental Table S2. Interestingly, there was little overlap between these top lists.

**Table 2:** Top altered proteins between control and MG samples

Biomarker	Control		Mild glaucoma		Fold change	t statistic	*p* value	FDR	Uniprot ID
	Mean	sd	Mean	sd					
LPS	1233.76	316.28	2892.49	434.45	2.34	−9.0496	7.87E-07	4.50E-04	O14896
CD45	2051.38	296.92	4363.33	533.06	2.13	−10.98	4.73E-07	3.25E-04	P08575
Gas1	1451.44	382.44	2748.5	252.46	1.89	−8.6294	2.55E-07	2.46E-04	P54826
TRPC6	1754.31	237.49	3062.42	389.78	1.75	−8.3349	4.36E-06	1.75E-03	Q9Y210
Fetuin B	2162.18	246.53	3755.37	406.19	1.74	−9.7498	9.58E-07	5.11E-04	Q9UGM5
Survivin	1032	78.6	1732.86	141.57	1.68	−12.5417	1.32E-07	1.51E-04	O15392
PAI-1	1954.93	167.3	3247.48	261.4	1.66	−12.1377	7.30E-08	9.73E-05	P05121
PIK3R1	4060.15	372.6	6608.07	597.83	1.63	−10.5292	3.81E-07	3.05E-04	P27986
EphA2	1253.84	229.86	2018.87	131.53	1.61	−8.8656	2.77E-07	2.46E-04	P29317
MATK	6973.61	1073.77	11,146.85	750.49	1.60	−9.6842	4.85E-08	7.76E-05	P42679
APN	2084.42	140.21	3262.8	161.34	1.57	−16.3102	1.62E-10	1.30E-06	P15144
VDB	1715.91	292.7	2672.07	272.14	1.56	−7.1618	2.64E-06	1.17E-03	P02774
Lyn	3221.62	428.98	4985.11	248.72	1.55	−10.9083	1.81E-08	4.83E-05	P07948
MINA	4891.09	471.75	7237.39	482.03	1.48	−10.3594	3.15E-08	6.30E-05	Q8IUF8
NF1	2994.07	193.97	4247.36	303.62	1.42	−10.1371	4.88E-07	3.25E-04	P21359
LTF	7110.74	741.97	9938.62	728.8	1.40	−8.1146	6.41E-07	3.95E-04	P02788
PSA-total	1860.2	130.97	2423.97	162.57	1.30	−7.9579	1.99E-06	9.93E-04	P07288
WDR4	1349.53	114.52	1678.5	85.58	1.24	−6.9709	3.20E-06	1.35E-03	P57081
ALBUMIN	205,095.55	38,442.66	64,437.98	40,880.77	0.31	7.4476	2.33E-06	1.10E-03	P02768
Thrombomodulin	262,637.13	36,630.88	61,347.23	35,543.15	0.23	11.7777	4.35E-09	1.74E-05	P07204

FDR, false discovery rate; sd, standard deviation.

**Table 3:** Top altered proteins between control and AG samples

Biomarker	Control		Advanced glaucoma		Fold change	t statistic	*p* value	FDR	Uniprot ID
	Mean	sd	Mean	sd					
PSP	3834.3	438.84	7436.49	1275.98	1.94	−9.1515	2.77E-07	1.53E-04	P08118
Tec	1744.63	305.21	3255.78	570.82	1.87	−7.9132	3.69E-07	1.64E-04	P42680
MINA	4891.09	471.75	8019.99	1135.9	1.64	−8.6855	2.75E-07	1.53E-04	Q8IUF8
SSEA-4	1960.78	198.56	3160.63	425.21	1.61	−8.7025	1.70E-07	1.28E-04	-
CABLES2	1563.6	147.1	2457.34	309.38	1.57	−8.8754	1.21E-07	1.18E-04	Q9BTV7
PAI-1	1954.93	167.3	3038.24	405.82	1.55	−8.4277	4.15E-07	1.75E-04	P05121
SSTR2	1450	165.88	2215.56	259.46	1.53	−8.3721	8.88E-08	1.18E-04	P30874
MTUS1	1873.49	137.15	2622.1	256.89	1.40	−8.7139	9.59E-08	1.18E-04	Q9ULD2
ALAS2	1855.57	161.27	2529.69	230.96	1.36	−8.031	1.32E-07	1.18E-04	P22557
BTC	5466.87	410.03	7360.16	728.92	1.35	−7.6601	4.86E-07	1.88E-04	P35070
TOPORS	1524.33	132.73	2044.77	147.42	1.34	−8.7068	3.27E-08	6.54E-05	Q9NS56
CAMK2N1	2415.52	116.29	3174.16	281.3	1.31	−8.5104	3.63E-07	1.64E-04	Q7Z7J9
SNAPC5	1370.72	121.8	1769.47	120.92	1.29	−7.671	2.86E-07	1.53E-04	O75971
BCDIN3D	1178.3	96.73	1519.17	107.82	1.29	−7.8109	1.76E-07	1.28E-04	Q7Z5W3
IL-13	10,644.04	563.4	13,044.46	890.34	1.23	−7.6757	3.30E-07	1.64E-04	P35225
NUP98	8692.95	442.5	5731.02	1059.37	0.66	8.8072	2.25E-07	1.50E-04	P52948
C5AR2	1924.96	224.79	1018.33	301.6	0.53	8.0662	1.10E-07	1.18E-04	Q9P296
ANGPTL2	4017.19	232.99	1925.22	517.22	0.48	12.5645	1.17E-09	7.54E-06	Q9UKU9
ALBUMIN	205,095.55	38,442.66	49,853.43	40,714.34	0.24	9.1809	1.53E-08	4.07E-05	P02768
Thrombomodulin	262,637.13	36,630.88	52,394.19	55,652.31	0.20	10.6151	1.89E-09	7.54E-06	P07204

FDR, false discovery rate; sd, standard deviation.

**Table 4:** Top altered proteins between MG and AG samples

Biomarker	Mild glaucoma		Advanced glaucoma		Fold change	t statistic	*p* value	FDR	Uniprot ID
	Mean	sd	Mean	sd					
HDDC3	3764.24	813.55	5535.66	973.15	1.47	−4.4059	3.90E-04	1.24E-01	Q8N4P3
DKK2	2301.28	389.19	3324.57	507.13	1.44	−5.0933	8.22E-05	1.20E-01	Q9UBU2
DEK	1906.29	254.86	2684.75	372.94	1.41	−5.545	2.92E-05	1.11E-01	P35659
DDX4	1970.16	281.84	2747.44	410.94	1.39	−5.0171	9.01E-05	1.20E-01	Q9NQI0
PCSK1	1849.17	254.66	2568.28	457.29	1.39	−4.5004	2.90E-04	1.24E-01	P29120
DGKG	1710.46	230.37	2344.08	331.61	1.37	−5.0412	8.59E-05	1.20E-01	P49619
CREG1	1813.3	280.28	2433.16	329.85	1.34	−4.5105	3.18E-04	1.24E-01	O75629
PTPRB	1778.49	227.32	2372.33	346.6	1.33	−4.6273	2.09E-04	1.24E-01	P23467
EIF2AK2	1614.3	229.93	2148.86	321.16	1.33	−4.3353	4.06E-04	1.24E-01	P19525
UBE2L6	1950.34	273.16	2586.83	318.05	1.33	−4.7766	1.84E-04	1.24E-01	O14933
TAFA1	1743.97	233.73	2233.13	248.77	1.28	−4.4681	3.98E-04	1.24E-01	Q7Z5A9
ZNF44	999.38	106.86	1264.4	130.83	1.27	−4.961	1.17E-04	1.24E-01	P15621
Fractalkine	4729.91	315.37	5781.75	397.8	1.22	−6.5715	4.33E-06	3.46E-02	P78423
IL-20	4208.15	411.58	5071.08	416.58	1.21	−4.5712	3.51E-04	1.24E-01	Q9NYY1
DSG4	1617.27	107.6	1943.51	196.15	1.20	−4.7824	1.59E-04	1.24E-01	Q86SJ6
GDF-15	5050.54	271.6	6030.43	527.18	1.19	−5.4454	4.18E-05	1.11E-01	Q99988
NrCam	4405.86	350.2	5135.12	347.5	1.17	−4.5764	3.59E-04	1.24E-01	Q92823
PSME2	3228.89	167.43	3761.44	355.86	1.16	−4.4917	3.38E-04	1.24E-01	Q9UL46
MIP-1b	5961.74	282.27	6852.02	550.91	1.15	−4.7418	1.84E-04	1.24E-01	P13236
IDO2	3014.41	126.14	3343.01	200.66	1.11	−4.4951	2.81E-04	1.24E-01	Q6ZQW0

FDR, false discovery rate; sd; standard deviation.

### Gene Ontology and Pathway Enrichment Analyses Identify Unique Biological Processes and Molecular Functions Associated With Disease Staging

**Fig. 2:**
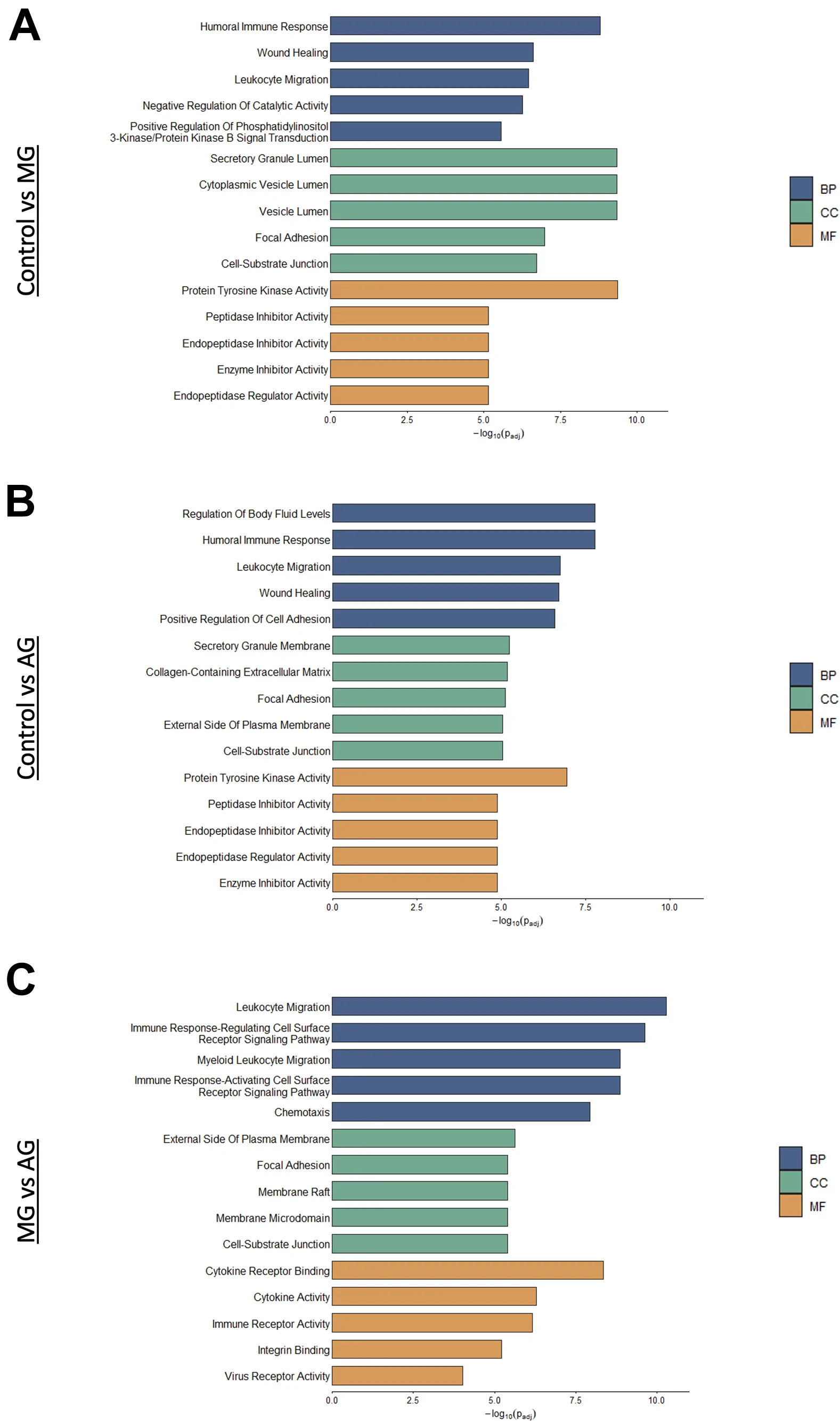
**GO analyses identifies unique pathways associated with disease staging.** Graphic displays of top significant GO analyses results associated with comparisons between (*A*) Control *versus* MG groups, (*B*) Control *versus* AG groups, and (*C*) MG *versus* AG groups. (BP; biological processes, CC; cellular components, MF; molecular functions).

To highlight pathological processes indicated by the proteomic data, gene ontology (GO) analyses were performed for each of the three comparisons. The comparison between Control and MG groups identified significantly enriched biological processes (BP), CC, and molecular functions (MF) involving wound healing (e.g. “wound healing”, “negative regulation of catalytic activity”, “focal adhesion”), extracellular matrix remodeling (e.g.; “peptidase inhibitor activity”, “enzyme inhibitor activity”, “endopeptidase regulator activity”, “cell substrate junction”), and adaptive immune responses (“humoral immune response”, “leukocyte migration”) (Fig. 2*A*). The comparison between Control and AG highlighted similar processes, components, and functions reflecting wound healing, extracellular matrix remodeling and adaptive immune responses (Fig. 2*B*). Intriguing, the comparison between MG and AG groups uniquely featured pathways involving in proinflammatory responses and signaling (Fig. 2*C*), including “regulation/activation of cell surface receptors”, “chemotaxis”, “leukocyte migration”, “cytokine receptor binding”, and “cytokine or immune receptor activity”. Together these results indicated that although both MG and AG feature wound healing and extracellular matrix remodeling pathways compared to non-glaucomatous controls, but the distinction between MG and AG is intriguingly focused on an altered proinflammatory signature and immune cell migration and chemotaxis.

**Figure S2:**
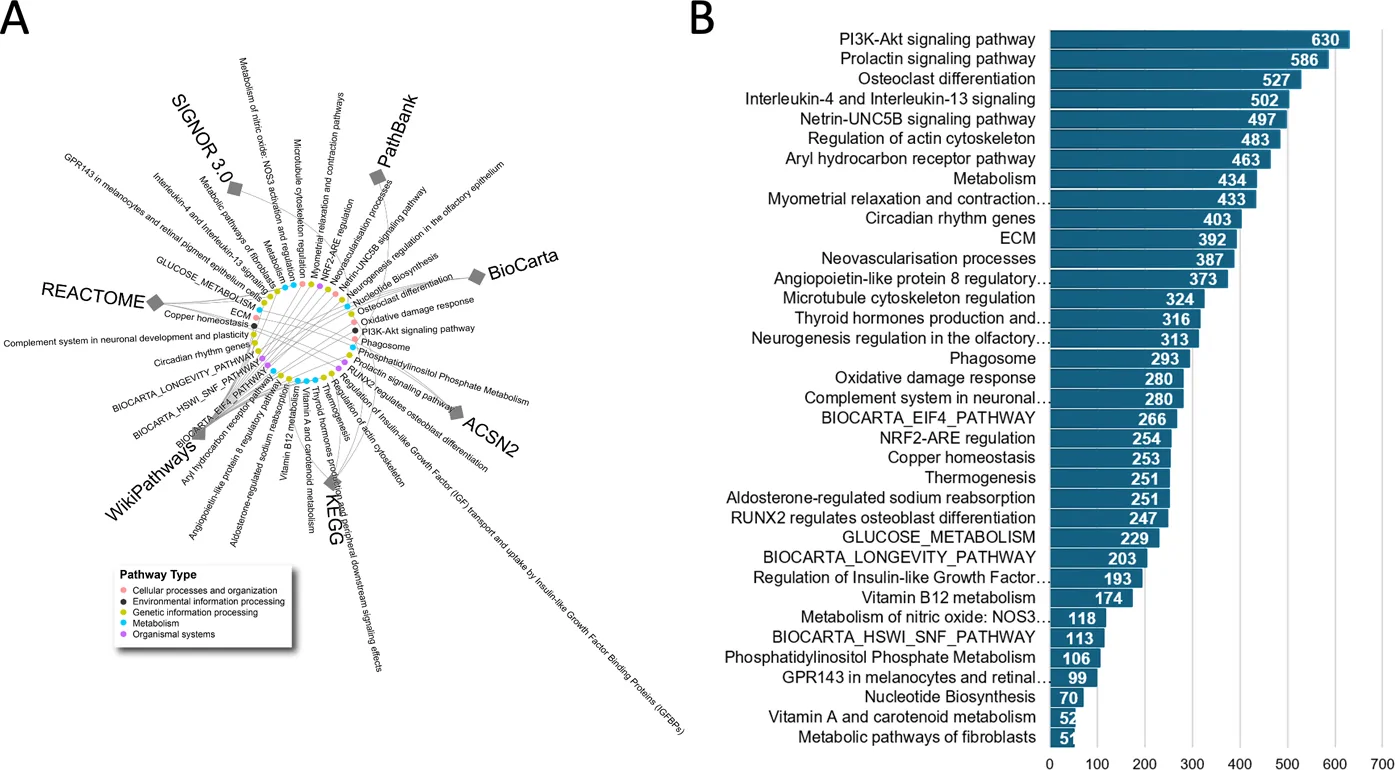
**Summary of MG vs AG results following PathDIP analyses.***A,* a visual representation of the pathway sources, pathways, and pathway type. *B,* the numbers of differential proteins detected per consolidated pathway. AG, advanced glaucoma group; MG, mild glaucoma group.

Additional pathway enrichment analysis using the pathDIP database revealed distinct biological signatures associated with each disease stage (Supplemental Tables S3–S5, Supplemental Fig. S2). In the MG *versus* control comparison, enriched pathways reflected structural remodeling and wound-healing responses, including focal adhesion, VEGFA–VEGFR2 signaling, RANKL/RANK, and neovascularization processes. Additional enrichment in BDNF, androgen receptor, and aryl hydrocarbon receptor pathways, suggest early tissue adaptation and metabolic stress responses. In AG *versus* MG, the pattern shifted toward inflammatory and immune activation, with strong enrichment of interleukin-4/13, complement, prolactin, and phagosome pathways. Co-enrichment of PI3K–Akt, osteoclast differentiation, and metabolic pathways indicates progressive immune-driven remodeling and metabolic adaptation accompanying disease progression. In the AG *versus* control comparison, enriched pathways showed global immune, structural, and metabolic dysregulation, including interleukin signaling, receptor tyrosine kinase, ECM organization, and cytokine–receptor interaction. Additional enrichment in angiogenic, oxidative, and metabolic pathways highlights cumulative remodeling in advanced disease.

### A Differential Protein Signature Correlates With Clinical Parameters

**Fig. 3:**
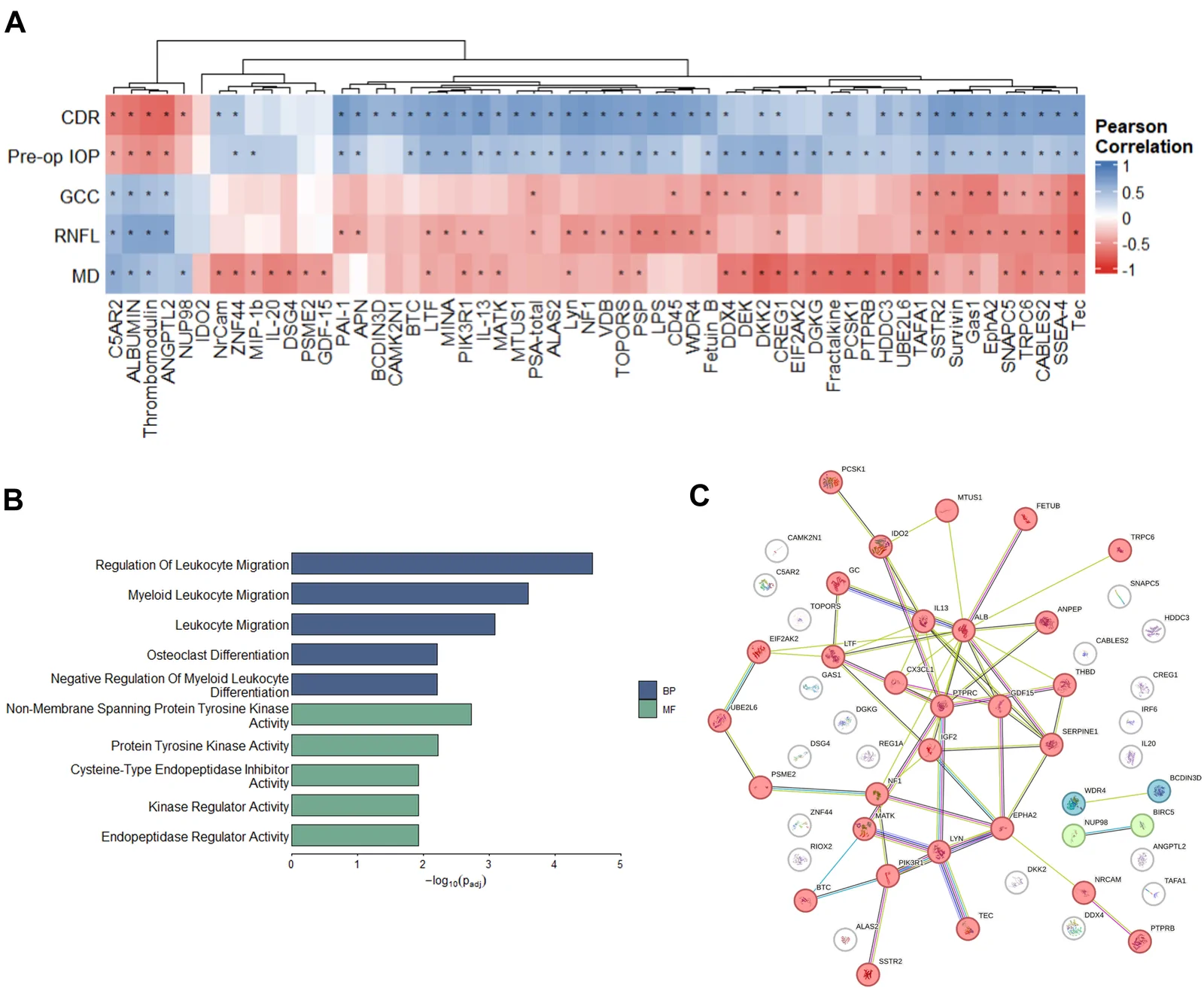
**A differential protein signature correlates with clinical parameters.***A*, the *top* 20 altered biomarkers from each group were compared and duplicates removed, to arrive at a unique signature of 56 biomarkers. This heatmap indicates the Pearson correlation for each marker across all patient samples for each of five relevant clinical parameters; CDR, Pre-op IOP, GCC thickness, RNFL thickness, and MD (∗*p* < 0.05 for each). *B*, significant GO analyses terms for this correlated protein signature. *C*, STRING analyses indicates a substantial interactome network among the signature proteins.

As we have access to the complete patient datasets, these proteomic analyses presented a unique opportunity to correlate patient biochemical signatures with multiple clinical parameters relevant to disease staging. We identified the top 20 most differentiated proteins from each of the three comparisons. After eliminating duplications, 56 unique proteins were identified that represented a proteomic signature that can reflect disease staging data. We then correlated the levels of these proteins for all patient samples against five key clinical parameters: CDR, pre-operative IOP, MD, RNFL thickness and GCC thickness. All parameters reflected substantial correlations in the expected direction with our proteomic signature (Fig. 3*A*). Interestingly, CDR and pre-operative IOP have the highest numbers of significant correlations (46 and 45 proteins, respectively), matching clinical parameters with most of the proteins in the signature. These were followed by MD (37 proteins correlated), RNFL thickness (31 proteins correlated) and GCC thickness (21 proteins correlated). We further performed GO analyses on this correlated signature, which revealed significant associations with immune cell migration (BP), and kinase and endopeptidase activities (MF) (Fig. 3*B*). Because many of these proteins have related inflammatory and immune functions, we also performed an interactome analyses using the STRING database, which revealed a substantial cellular signaling network (Fig. 3*C*). Therefore, this small protein signature correlating with clinical endpoints exhibits pathway and interactome networks closely tied to inflammation and immune cell function and migration.

### Identification of TRPC6 as a Novel Biomarker Associated With Increased Glaucoma Staging

**Fig. 4:**
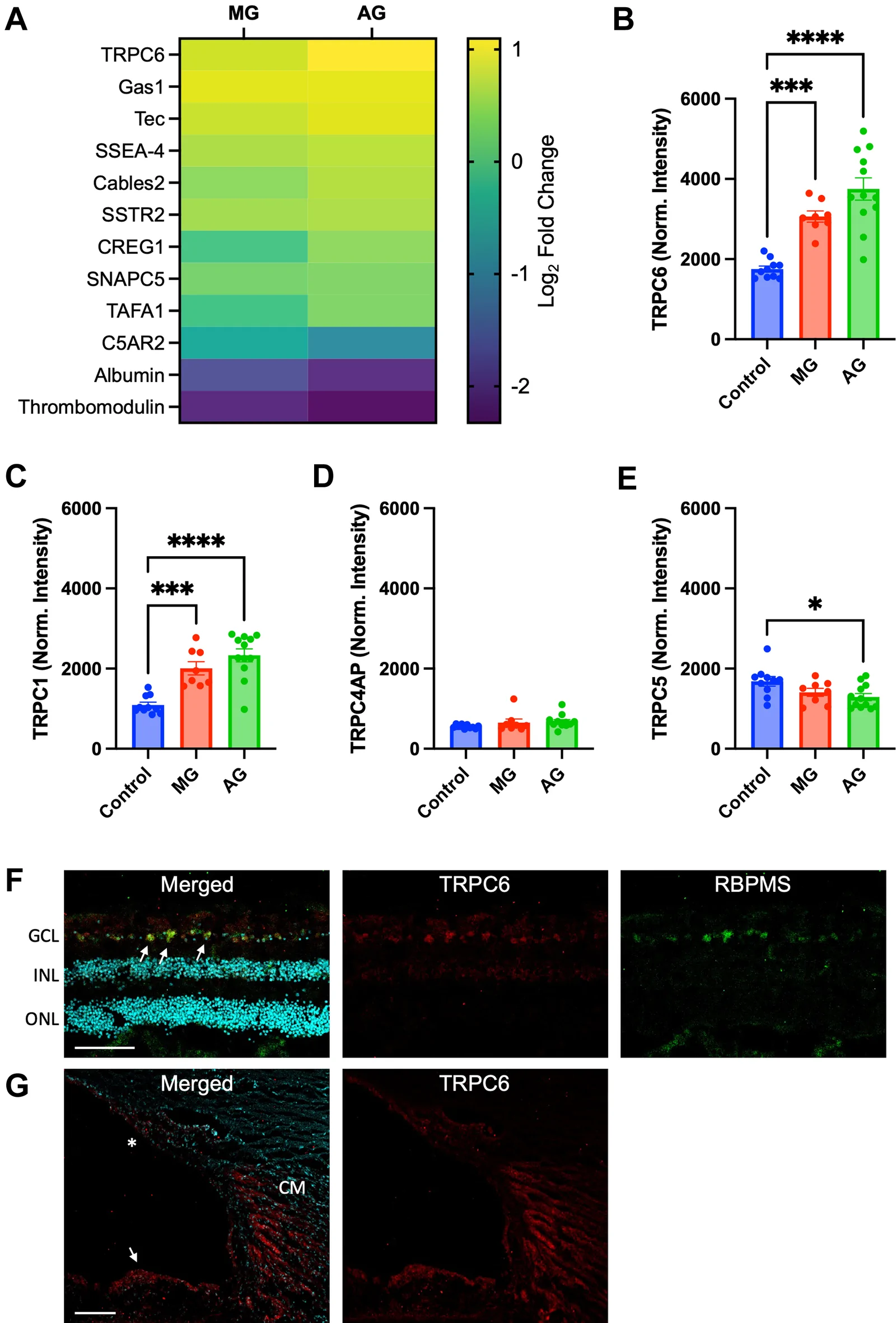
**TRPC6 levels correlate strongly with all clinical parameters and is expressed at the front and back of the eye.***A*, a heatmap showing log_2_ fold change for the proteins that significantly correlated with all clinical parameters. The most elevated with glaucoma progression is TRPC6. *B*, relative levels of TRPC6 in the three patient groups indicates progressive increases from control to MG and AG (∗∗∗*p* < 0.005, ∗∗∗∗*p* < 0.001, bars are SE). *C–E*, TRPC1 shows a similar increase, but not TRPC4AP or TRPC5 (∗*p* < 0.05, ∗∗∗*p* < 0.005, ∗∗∗∗*p* < 0.001, bars are SE). *F*, human retinal staining for TRPC6 (*red*) shows a positive signal in the inner retina that colocalizes with the RGC marker, RBPMS (*green*), in comparison to DAPI staining to highlight morphology (*blue*; merged). (Scale bar indicates 200 μm). *G*) Antibody staining of human anterior angle tissues for TRPC6 (*red*) shows a positive signal in the iris epithelium (*arrow*), TM (*asterisk*), and ciliary muscle (CM), in comparison to DAPI staining to highlight morphology (*blue*; merged). (Scale bar indicates 100 μm).

**Figure S3:**
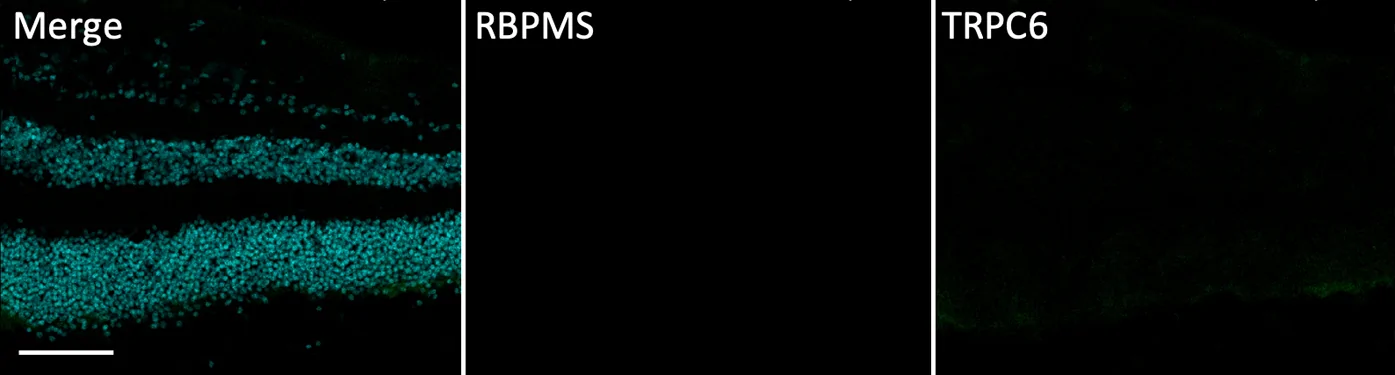
**No primary control for TRPC6 staining.** No primary antibody was included on 1 section to control for nonspecific secondary antibody staining.

**Figure S4:**
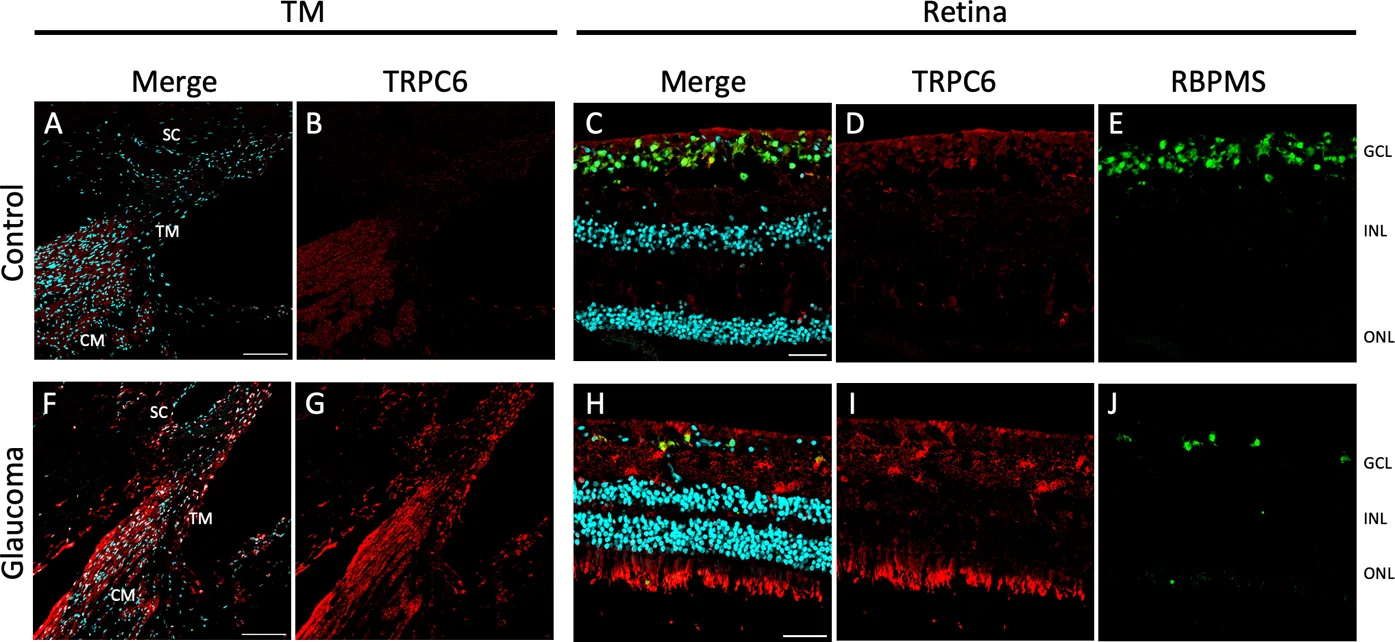
**TRPC6 staining of human glaucomatous anterior segment and retinal tissues.***A* and *B,* representative image of the anterior segment of a healthy human eye, stained with antibodies directed to TRPC6 (*red*), compared with DAPI (*blue*). *C*–*E,* representative image of the retina of a healthy human eye, stained for TRPC6 (*red*) and RBPMS (*green*), labeling the RGC and nerve fiber layers. *F* and *G,* corresponding image of the anterior segment of a glaucomatous human eye showing increased TRPC6 staining (*red*), which highlights increased signal in the Schlemm’s canal (SC), trabecular meshwork (TM), and ciliary muscle (CM). *H*–*J,*. Corresponding image of the glaucomatous retina with increased TPC6 staining (*red*) in the GCL and INL layers. GCL, ganglion cell layer; INL, inner nuclear layer; ONL, outer nuclear layer. Scale bars represent 50 µm (*A*–*E*), 100 µm (*F*–*J*).

To further prioritize potential biomarkers we isolated the proteins that significantly correlated with *all five* clinical endpoints assessed, resulting in a list of 12 candidates (Fig. 4*A*). Of these candidates the most differentially regulated was TRPC6, which was strongly and progressively increased in MG and AG samples (Fig. 4*B*). Intriguingly, TRPC6 is a cation channel linked to a variety of mechanical and chemical stimuli, often in the context of leukocyte migration and recruitment (31, 32, 33), and fibrosis (34, 35, 36), which is consistent with our pathway analyses data. As TRPC6 is a member of a larger subfamily, we investigated the presence of other TRPC family members in our proteomic data. TRPC1 is often closely associated with TRPC6 (37, 38), and was also elevated in our patient samples (Fig. 4*C*). However, other TRP channels and interactors, including TRPC5, and TRPC4-associated protein (TRPC4AP), were not increased (Fig. 4, *D* and *E*), demonstrating a degree of specificity. In addition to its immune and fibrosis roles, TRPC6 has been recently studied in the context of neuronal function and RGC degeneration (39, 40, 41). Antibody staining for TRPC6 in human retinal tissues revealed prominent signal enriched in retinal ganglion cells (Fig. 4*F*, no-primary control in Supplemental Fig. S3), raising the possibility of a local retinal origin for this signal. However, our staining for TRPC6 in human anterior eye tissues also revealed signal in the iris epithelium, TM, and ciliary muscle (Fig. 4*G*). We were able to obtain a few matched eye sections from glaucoma patients and two healthy controls that suggested increased TRPC6 staining in diseased TM and inner retina (Supplemental Fig. S4). Together, these data identify TRPC6 as a potential novel AH biomarker for glaucoma progression.

## Discussion

POAG remains a leading cause of permanent visual impairment worldwide, especially in an aging population. Identifying biochemical profiles at different stages of POAG are crucial to better understand the mechanisms of its pathogenesis and progression, and uncover novel disease biomarkers. To our knowledge, this is largest scale proteomic study of POAG patient samples to date that assessed different disease stages, interrogating a comprehensive panel of 8000 AH proteins.

**Figure S5:**
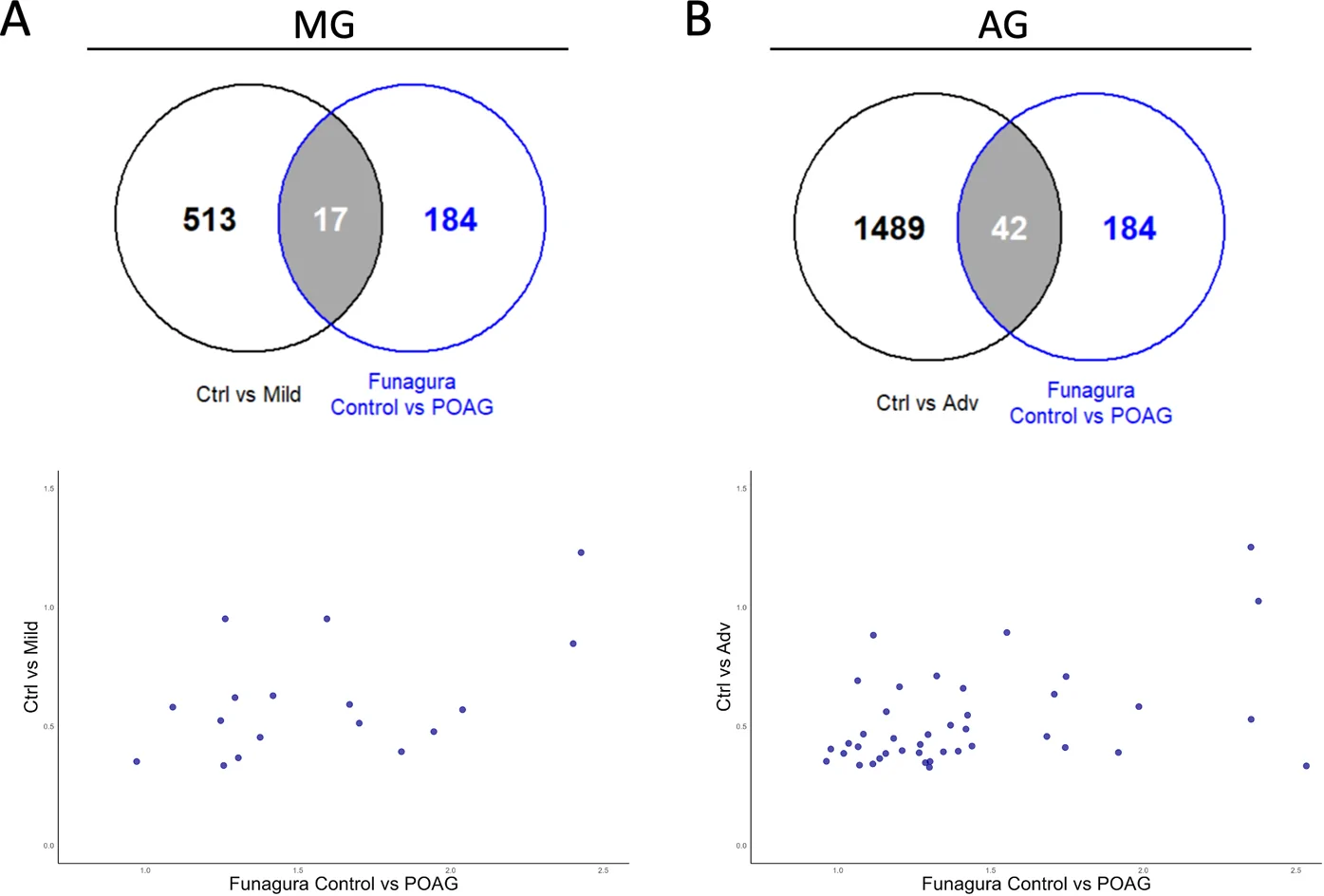
**Comparison between study data and an external proteomic signature.***A,* significantly upgregulated proteins from our control:MG data were compared to the broad POAG signature from Funagura et al, with an overlap of 17 proteins and overall positive correlation. *B,* significantly upregulated proteins from our control:AG data were compared to the broad POAG signature with an overlap of 42 proteins and overall positive correlation. POAG, primary open-angle glaucoma; AG, advanced glaucoma group; MG, mild glaucoma group.

A majority of previous clinical proteomic studies have focused on a limited number of molecules, primarily multiplexed analyses of cytokines and chemokines (18, 19, 20, 21, 42, 43). More recently, several groups have reported medium- or large-scale proteomic analyses (17, 44, 45, 46, 47). However, as far as we are aware, all of these studies to date have used broad categorizations of disease *versus* control groups, without consideration of disease staging or progression. Perhaps the most comparable study to ours was recently reported by Funagura *et al* (44), which used large-scale array-based proteomics to assess patients with a general classification of POAG. These data are not directly comparable to our mild *versus* advanced disease samples. Still, when comparing their proteomic POAG signature to our control:AG groups, we found a substantial overlap of 42/184 (23%) significantly upregulated proteins with a positive correlation (Supplemental Fig. S4), including two of our top disease stage-correlating hits, GDF15 and BTC. In comparison, our control: MG data saw an overlap of only 17/184 (9%) significantly upregulated proteins, also with a positive correlation, possibly because there were fewer mild glaucoma patients included in their analyses (Supplemental Fig. S5). Unfortunately, the array used in their study did not assess for TRPC6 or TRPC1. Thus, our approach enabled a broad interrogation of early *versus* advanced POAG, facilitating the identification of new and previously unknown shifts related to disease staging. These foundational data provide insights into the possible mechanisms driving POAG progression. Notably, our data clearly distinguish between controls and mild glaucoma patients, indicating substantial differences in biochemical signatures. In comparison, a prior POAG patient sample analysis based on clinical OCT parameters did not provide as clean a separation (48). Our analyses also revealed surprisingly close associations between aqueous fluid biomarkers and retinal disease measures of neurodegeneration (CDR, RNFL), and functional vision loss (MD).

The comprehensive nature of this study facilitated multiple analytical approaches, revealing intriguing insights. Our comparison between controls and MG groups generated a surprisingly robust biochemical signature associated with mild disease. Pathways involved in wound healing, extracellular matrix (ECM) remodeling and adaptive immune response stood out prominently. These observations are consistent with prior evidence for ECM and complement dysregulation in trabecular meshwork dysfunction during glaucoma pathogenesis (49, 50). Remarkably, even with relatively small sample sizes, there were also differences in proteomic profiles between early *versus* advanced glaucoma stages. For example, GO comparisons between MG *versus* AG were substantively different from control *versus* MG, with significant molecular functions and biological processes highlighting the role of proinflammatory and cytokine signaling. It is not clear whether these results reflect a causal relationship with advanced disease, or are a consequence of ongoing pathological processes. Yet, our results are consistent with the concept of an inflammatory switch underlying advanced trabecular meshwork dysfunction to drive disease progression (51). Supporting this idea, comprehensive pathway analysis using pathDIP revealed a molecular transition to broad immune and inflammatory activation in advanced disease in addition to ongoing ECM remodeling and wound healing. These interconnected pathways highlight glaucoma progression as a cumulative systems-level process rather than a single pathway-driven event.

Finally, these proteomic data presented a rare opportunity for comparison with corresponding clinical parameters, utilizing the entire sample set to assess for correlations. As far as we are aware, this is the first large-scale proteomic study to make this type of comparison. Intriguingly, our analyses revealed that many of the top differential AH proteins correlated significantly with multiple retinal disease parameters, such as CDR, RNFL thickness, and MD, in addition to IOP. These results suggest that biochemical signatures in AH may reflect neurological disease processes, rather than just ocular hypertension. Pathways and interactome analyses of these top correlated proteins revealed a tight network of proteins involved in inflammation and systemic immune cell function, particularly migration and chemotaxis, echoing the results of our earlier broad pathway analyses for mild to advanced disease. This pattern is consistent with recently reported plasma proteomics by Langbol *et al*, comparing patients with POAG and normal tension glaucoma, similarly showing an elevated immune signature (47). Notable proteins in this list include GDF15, a TGF-β superfamily member that mediates cellular stress signaling and has been recently reported to be elevated in glaucomatous AH and serum, and correlates with disease severity (44, 52, 53, 54). Similarly, PAI-1 is an important inhibitor of fibrinolysis, and elevated levels have been observed in glaucomatous AH (55) and are associated with TGF-β-induced outflow models of TM cell dysfunction and fibrosis (56, 57, 58).

By ranking the shortlist of proteins that significantly correlated with all clinical parameters, we were able to further identify biomarkers that most robustly reflected clinical disease staging. This list highlighted soluble TRPC6 as a novel biomarker that corresponds closely with POAG disease stage. TRPC channels are a subfamily of voltage-independent transmembrane cation channels with functions linked to the sensing of a variety of mechanical, chemical, and oxidative/metabolic stimuli, often in the context of leukocyte migration and recruitment (31, 32, 33), (cardiac) fibrosis (34, 35, 36), and neuronal function or neurodegeneration (39, 40). Increasing evidence also indicates that TRP family members or other transmembrane proteins can be shed from extracellular vesicles, such as exosomes, microvesicles, or dying cells (59, 60, 61).

Elevated TRPC6 has recently been implicated in several glaucoma-related studies, often assembling with TRPC1 to form heteromeric channels (37, 38). A study of 80 POAG patients found significantly elevated TRPC6 expression in peripheral blood leukocytes, which was notably not affected by IOP-lowering medications (62). Also, TRPC6 protein was reported in human TM and outflow tissues (63), along with other TRPC channels (37, 64). Most of the reported experimental work on TRPC6 in the eye has been in the retina and ONH. TRPC6 levels were elevated in rat RGCs in an ischemia/reperfusion model where it was proposed to contribute to metabolic stress, and inhibition of this signal was protective (41). Similarly, increased TRPC6 expression and function have been reported in astrocytes and lamina cribrosa cells in the ONH, and Müller glia (65, 66, 67). Both TRPC6 and TRPC1 levels were similarly elevated in our AH samples, but not TRPC5 or TRPC4AP, demonstrating a specific response. There are multiple candidate sources for TRPC6 in AH. Because TRPC6 has mostly been studied in the context of the inner retina and CNS, the concept of a retinal POAG biomarker appearing in AH is exciting. Alternatively, our data confirmed TRPC6 immunostaining in human outflow tissues, and so this signal could indicate a role in mechanosensing elevated IOP. A third, and perhaps the most likely scenario, is for TRPC6 to be shed from peripheral leukocytes as the disease progresses, which is most consistent with our overall pathway signature. Substantial additional work will be required to disentangle these possibilities and clarify the functional role of elevated TRPC6 in POAG staging and progression.

There are a few important limitations to note in our study. First, these data are cross-sectional and do not directly address changes underlying glaucoma progression. Instead, we examined the proteomic signature at three different states, identified differential proteins, and deduced the pathways and processes that varied between each comparison. Secondly, as seen in the PCA plot (Fig. 1*A*), there were two apparent outliers in our samples. Sample AG10 came from a patient with advanced glaucoma, but their proteomic signature was broadly similar to those in the control group. Meanwhile, MG1, coming from a patient with mild glaucoma, surprisingly did not share general molecular similarity with either the control or glaucoma groups. For both outliers, we methodically reviewed our process, from sample collection to data analysis and did not uncover any irregularities or cause for exclusion. Therefore, we included these data in our analyses. Lastly, as an initial study, our sample size is relatively small, and variables such as sex or race were not powered for analyses. Some patients originally assigned to the MG group progressed rapidly and reached advanced status by the time of their surgeries, requiring a reclassification. Yet, even so clear and significant differences were identified between the group data sets, supporting the future use of our approach and methodology to uncover unbiased proteomic changes in AH samples.

In summary, our large-scale proteomic study of aqueous humor from patients at different stages of POAG offers novel insights into the biochemical alterations associated with disease development and progression. By expanding the scope beyond targeted cytokine or chemokine analyses, we identified key pathways - particularly those related to wound healing, extracellular matrix remodeling, adaptive immunity and inflammation, and identified novel biomarkers. These pathways appear intricately linked to POAG pathophysiology and staging. Our findings lay important groundwork for future longitudinal and mechanistic studies. Ultimately, these insights inform the discovery of novel biomarkers and therapeutic targets aimed at halting or slowing glaucoma progression.

## Data Availability

Complete proteomic data for all samples are attached as a supplemental file (Supplemental Table S1).

## Supplemental data

This article contains supplemental data.

## Conflict of interest

The authors declare that they have no conflicts of interest with the contents of this article.
